# Ethyl 5-hy­droxy-6-oxo-4-phenyl-5,6-dihydro-4*H*-cyclo­penta­[*b*]thio­phene-5-carboxyl­ate

**DOI:** 10.1107/S1600536811037032

**Published:** 2011-09-30

**Authors:** C. John McAdam, Stephen C. Moratti, Jim Simpson, Zheng Shi

**Affiliations:** a Department of Chemistry, University of Otago, PO Box 56, Dunedin, New Zealand

## Abstract

In the title mol­ecule, C_16_H_14_O_4_S, the dihydro­cyclo­penta­thio­phenone ring system is almost planar, with an r.m.s. deviation of 0.060 Å from the best fit plane through all nine non-H atoms. The cyclo­penta­none ring adopts a severely flattened envelope conformation with the C atom carrying the OH and ethylcarboxylate substituents at the flap. This atom lies only 0.185 (3) Å from the plane through the other four C atoms. The phenyl substituent is inclined at 43.37 (5)° to the dihydro­cyclo­penta­thio­phenone mean plane. In the crystal, mol­ecules are linked by pairs of O—H⋯O hydrogen bonds, forming inversion dimers with *R*_2_^2^(10) ring motifs. Weak C—H⋯O hydrogen bonds also link mol­ecules into chains along *c*, while an approximately orthogonal set of C—H⋯O contacts form chains along *b*, resulting in layers lying parallel to (100). Inversion dimers also form through weaker *R*_2_^2^(12) C—H⋯S contacts, which combine with C—H⋯O contacts to form stacks along *b*.

## Related literature

For details of conducting thio­phene polymers, see: Anquetil *et al.* (2003 ▶). For related structures, see: Bonini *et al.* (2004 ▶); Chang *et al.* (2004 ▶). For details of the Cambridge Structural Database, see: Allen (2002 ▶). For hydrogen-bond motifs, see: Bernstein *et al.* (1995 ▶). For standard bond lengths, see Allen *et al.* (1987 ▶). For the preparation of a precursor used in the synthesis, see: Yang (2009 ▶).
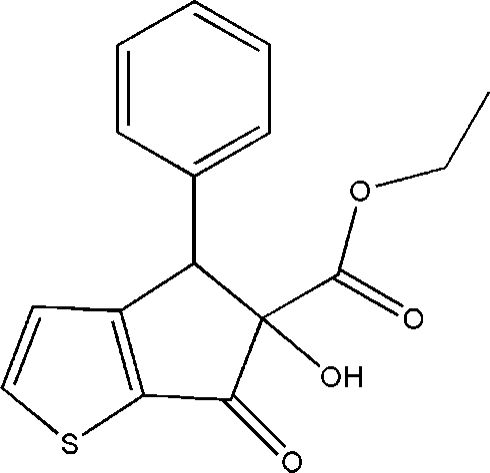


## Experimental

### 

#### Crystal data

C_16_H_14_O_4_S
*M* *_r_* = 302.33
Triclinic, 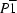

*a* = 7.7407 (4) Å
*b* = 9.1005 (4) Å
*c* = 10.5035 (5) Å
α = 84.840 (3)°
β = 80.929 (3)°
γ = 88.086 (3)°
*V* = 727.56 (6) Å^3^
*Z* = 2
Mo *K*α radiation
μ = 0.24 mm^−1^
*T* = 91 K
0.45 × 0.35 × 0.25 mm

#### Data collection

Bruker APEXII CCD area-detector diffractometer
Absorption correction: multi-scan (*SADABS*; Bruker, 2011 ▶) *T*_min_ = 0.672, *T*_max_ = 0.745
13007 measured reflections
2584 independent reflections
2233 reflections with *I* > 2σ(*I*)
*R*_int_ = 0.035

#### Refinement

*R*[*F* ^2^ > 2σ(*F* ^2^)] = 0.032
*wR*(*F* ^2^) = 0.079
*S* = 1.07
2584 reflections
194 parameters
H atoms treated by a mixture of independent and constrained refinement
Δρ_max_ = 0.30 e Å^−3^
Δρ_min_ = −0.26 e Å^−3^

### 

Data collection: *APEX2* (Bruker, 2011 ▶); cell refinement: *APEX2* and *SAINT* (Bruker, 2011 ▶); data reduction: *SAINT*; program(s) used to solve structure: *SHELXS97* (Sheldrick, 2008 ▶) and *TITAN2000* (Hunter & Simpson, 1999 ▶); program(s) used to refine structure: *SHELXL97* (Sheldrick, 2008 ▶) and *TITAN2000*; molecular graphics: *SHELXTL* (Sheldrick, 2008 ▶) and *Mercury* (Macrae *et al.*, 2008 ▶); software used to prepare material for publication: *SHELXL97*, *enCIFer* (Allen *et al.*, 2004 ▶), *PLATON* (Spek, 2009 ▶) and *publCIF* (Westrip 2010 ▶).

## Supplementary Material

Crystal structure: contains datablock(s) global, I. DOI: 10.1107/S1600536811037032/su2314sup1.cif

Structure factors: contains datablock(s) I. DOI: 10.1107/S1600536811037032/su2314Isup2.hkl

Supplementary material file. DOI: 10.1107/S1600536811037032/su2314Isup3.cml

Additional supplementary materials:  crystallographic information; 3D view; checkCIF report

## Figures and Tables

**Table 1:** Hydrogen-bond geometry (Å, °)

*D*—H⋯*A*	*D*—H	H⋯*A*	*D*⋯*A*	*D*—H⋯*A*
O6—H6⋯O14	0.82 (2)	2.27 (2)	2.6705 (16)	110.8 (16)
O6—H6⋯O5^i^	0.82 (2)	2.06 (2)	2.7973 (16)	150.5 (19)
C1—H1⋯O14^ii^	0.95	2.56	3.157 (2)	121
C11—H11⋯O5^iii^	0.95	2.52	3.386 (2)	152
C15—H15*B*⋯O6^iv^	0.99	2.27	3.206 (2)	157
C7—H7⋯S1^v^	1.00	2.97	3.7855 (16)	139
C12—H12⋯O14^vi^	0.95	2.61	3.497 (2)	157

Symmetry codes: (i) 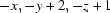; (ii) 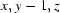; (iii) 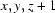; (iv) 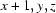; (v) 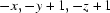; (vi) 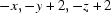.

**Fig. 1.**
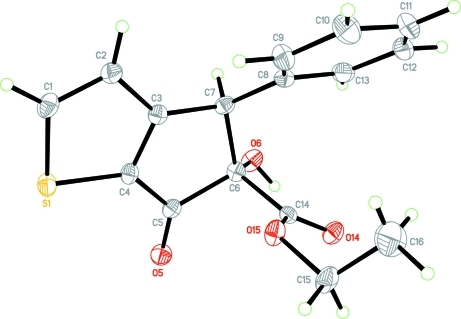
The molecular structure of the title molecule, with numbering scheme and dispacement ellipsoids drawn at the 50% probability level.

**Fig. 2.**
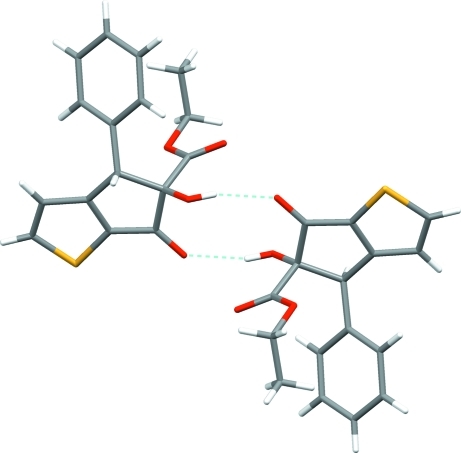
A view of the inversion dimers of the title compound formed by O—H···O hydrogen bonds [dashed cyan lines].

**Fig. 3.**
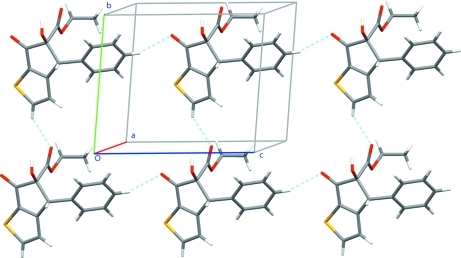
A view of the formation of layers of the title compound lying parallel to the (100) plane. The C—H···O interactions are illustrated by dashed cyan lines.

**Fig. 4.**
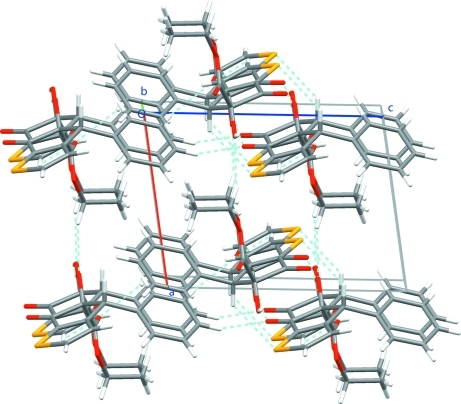
The crystal packing of the title compound viewed along the b-axis, showing the C—H···S and C—H···O interactions [dashed cyan lines].

## supplementary crystallographic information

### Comment

There is currently much interest in research into conducting thiophene polymers (Anquetil *et al.*, 2003). Our interest in molecular actuators based on this chemistry led to the isolation of the previously unreported title compound.

In the molecular structure of the title molecule (Fig. 1), the flap atom of the severely flattened C3···C7 ring carries both hydroxy and ethylcarboxylate substituents. Atom C6 lies 0.185 (3) Å from the mean plane through atoms (C3-C5,C7) and this plane is inclined at 4.43 (8) ° to the thiophene ring (S1,C1-C4) mean plane. As a result the dihydrocyclopenta-thiophene-one ring system is also reasonably planar with a r.m.s. deviation of only 0.060 Å from the plane through all 9 non-hydrogen atoms. The C7 atom of the cyclopentane ring carries a phenyl substituent that subtends a dihedral angle of 43.37 (5) ° to the dihydrocyclopentathiophene plane. The Cambridge Crystal Structure Database (CSD, Version 5.32, last update Aug. 2011; Allen, 2002) reveals only three structures involving dihydrocyclopenta-thiophene-one ring systems (Bonini *et al.*, 2004; Chang *et al.*, 2004). In the title molecule the bond distances are normal (Allen *et al.*, 1987) and similar to those reported for these similar molecules.

In the crystal O6–H6···O5 hydrogen bonds form inversion dimers with *R*^2^_2_(10) ring motifs, (Bernstein *et al.*, 1995), Fig 2. Chains form along *c* due to weak C11–H11···O5 contacts while C1–H1..O14 contacts form chains at roughly 90 ° to these along *b*. These contacts combine to generate layers lying parallel to the (100) plane, Fig 3. Inversion dimers also form through weaker *R*^2^_2_(12) C7–H7···S1 contacts, and together with C12..H12···O14 hydrogen bonds lead to stacks along *b*, Fig 4. Additional C15..H15···O6 contacts further stabilize the packing.

### Experimental

The precursor thiophene, ethyl 6-oxo-4-phenyl-5,6-dihydro-4*H*-cyclopenta- thiophene-5-carboxylate (EOPDCTC), was prepared by a literature procedure (Yang, 2009). EOPDCTC (315 mg, 1.1 mmol) and potassium tertiary butoxide (289 mg, 2.6 mmol) were dissolved in 2-propanol (5 ml) and iodine (280 mg, 1.1 mmol) in 2-propanol (5 ml) added dropwise under a N_2_ atmosphere. After stirring at room temperature for 24 hrs, the solvent was removed under reduced pressure. The residues were redissolved in EtOAc, washed with saturated sodium thiosulfate and distilled water then dried over MgSO_4_. The crude product was purified by silica gel column chromatography with CH_2_Cl_2_/EtOAc (10%) to give a brown solid in 17% yield. X-ray quality crystals were obtained from a CH_2_Cl_2_ solution of the title compound layered with hexane. *M*.p 404 K. HRMS (+ve ESI) m/*z* calc for C_16_H_13_O_4_SNa 324.0427, found 325.1152. Spectroscopic data for the title compound are available in the archived CIF.

### Refinement

The H atom of the OH group was located in a difference Fourier map and its coordinates were refined with *U*_iso_ = 1.5*U*_eq_(O). All C-bound H atoms were included in calulated positions and refined using a riding model: d(C—H) = 0.95, 1.00, 0.99 and 0.98 Å, for aromatic, methine, methylene and methyl H-atoms, respectively, with *U*_iso_ = k × *U*_eq_(C), where k = 1.5 for methyl H atoms, and k = 1.2 for all other H atoms.

### Crystal data

**Table:**

C_16_H_14_O_4_S	*Z* = 2
*M**_r_* = 302.33	*F*(000) = 316
Triclinic, *P*1	*D*_x_ = 1.380 Mg m^−^^3^
Hall symbol: -P 1	Mo *K*α radiation, λ = 0.71073 Å
*a* = 7.7407 (4) Å	Cell parameters from 4122 reflections
*b* = 9.1005 (4) Å	θ = 2.7–24.9°
*c* = 10.5035 (5) Å	µ = 0.24 mm^−^^1^
α = 84.840 (3)°	*T* = 91 K
β = 80.929 (3)°	Block, colourless
γ = 88.086 (3)°	0.45 × 0.35 × 0.25 mm
*V* = 727.56 (6) Å^3^	

### Data collection

**Table:**

Bruker APEXII CCD area-detector diffractometer	2584 independent reflections
Radiation source: fine-focus sealed tube	2233 reflections with *I* > 2σ(*I*)
graphite	*R*_int_ = 0.035
φ and ω scans	θ_max_ = 25.2°, θ_min_ = 2.7°
Absorption correction: multi-scan (*SADABS*; Bruker, 2011)	*h* = −9→9
*T*_min_ = 0.672, *T*_max_ = 0.745	*k* = −10→10
13007 measured reflections	*l* = −12→12

### Refinement

**Table:**

Refinement on *F*^2^	Primary atom site location: structure-invariant direct methods
Least-squares matrix: full	Secondary atom site location: difference Fourier map
*R*[*F*^2^ > 2σ(*F*^2^)] = 0.032	Hydrogen site location: inferred from neighbouring sites
*wR*(*F*^2^) = 0.079	H atoms treated by a mixture of independent and constrained refinement
*S* = 1.07	*w* = 1/[σ^2^(*F*_o_^2^) + (0.0322*P*)^2^ + 0.3338*P*] where *P* = (*F*_o_^2^ + 2*F*_c_^2^)/3
2584 reflections	(Δ/σ)_max_ < 0.001
194 parameters	Δρ_max_ = 0.30 e Å^−^^3^
0 restraints	Δρ_min_ = −0.26 e Å^−^^3^

### Special details

**Table:**

Experimental. Spectroscopic data for the title compound: ^1^H NMR (δ p.p.m., CDCl_3_, 400 MHz): 8.08 (1*H*, d, J = 4.8 Hz, C*H*S), 7.35–7.28 (5*H*, m, phenyl *H*), 7.16 (1*H*, d, J = 4.8 Hz, C*H*CHS), 4.73 (1*H*, s, C*H*C_6_H_5_), 4.37 (1*H*, s, O*H*), 3.82 & 3.62 [2 *x* (1*H*, m, C*H*_2_)], 0.85 (3*H*, t, J = 7 Hz, C*H*_3_). IR ν(CO) 1737, 1703 cm^-1^ .
Geometry. All e.s.d.'s (except the e.s.d. in the dihedral angle between two l.s. planes) are estimated using the full covariance matrix. The cell e.s.d.'s are taken into account individually in the estimation of e.s.d.'s in distances, angles and torsion angles; correlations between e.s.d.'s in cell parameters are only used when they are defined by crystal symmetry. An approximate (isotropic) treatment of cell e.s.d.'s is used for estimating e.s.d.'s involving l.s. planes.
Refinement. Refinement of *F*^2^ against ALL reflections. The weighted *R*-factor *wR* and goodness of fit *S* are based on *F*^2^, conventional *R*-factors *R* are based on *F*, with *F* set to zero for negative *F*^2^. The threshold expression of *F*^2^ > σ(*F*^2^) is used only for calculating *R*-factors(gt) *etc*. and is not relevant to the choice of reflections for refinement. *R*-factors based on *F*^2^ are statistically about twice as large as those based on *F*, and *R*- factors based on ALL data will be even larger.

### Fractional atomic coordinates and isotropic or equivalent isotropic displacement parameters (Å^2^)

**Table:**

	*x*	*y*	*z*	*U*_iso_*/*U*_eq_	
S1	0.31491 (5)	0.47012 (4)	0.41577 (4)	0.01868 (13)	
C1	0.2877 (2)	0.33654 (18)	0.54308 (16)	0.0206 (4)	
H1	0.3238	0.2366	0.5349	0.025*	
C2	0.2095 (2)	0.38688 (18)	0.65715 (16)	0.0185 (4)	
H2	0.1850	0.3271	0.7365	0.022*	
C3	0.1697 (2)	0.53999 (17)	0.64162 (15)	0.0149 (3)	
C4	0.2184 (2)	0.59827 (17)	0.51632 (15)	0.0155 (3)	
C5	0.1669 (2)	0.75165 (17)	0.49802 (15)	0.0154 (3)	
O5	0.17464 (15)	0.83394 (12)	0.39918 (10)	0.0204 (3)	
C6	0.0911 (2)	0.79820 (17)	0.63548 (15)	0.0152 (3)	
O6	−0.07237 (14)	0.86931 (13)	0.63575 (12)	0.0203 (3)	
H6	−0.064 (3)	0.959 (2)	0.6268 (19)	0.030*	
C7	0.0721 (2)	0.65116 (17)	0.72591 (15)	0.0158 (3)	
H7	−0.0542	0.6251	0.7382	0.019*	
C8	0.1173 (2)	0.66251 (18)	0.86036 (15)	0.0174 (4)	
C9	0.2418 (2)	0.57266 (19)	0.91240 (16)	0.0228 (4)	
H9	0.3044	0.5001	0.8629	0.027*	
C10	0.2752 (3)	0.5886 (2)	1.03705 (17)	0.0293 (4)	
H10	0.3582	0.5247	1.0728	0.035*	
C11	0.1892 (3)	0.6958 (2)	1.10879 (17)	0.0292 (4)	
H11	0.2145	0.7074	1.1929	0.035*	
C12	0.0658 (3)	0.78667 (19)	1.05765 (17)	0.0272 (4)	
H12	0.0067	0.8614	1.1064	0.033*	
C13	0.0281 (2)	0.76864 (18)	0.93510 (16)	0.0216 (4)	
H13	−0.0595	0.8293	0.9017	0.026*	
C14	0.2214 (2)	0.90456 (17)	0.67132 (14)	0.0147 (3)	
O14	0.18207 (15)	1.02944 (12)	0.69500 (11)	0.0212 (3)	
O15	0.38114 (14)	0.84388 (12)	0.66685 (11)	0.0200 (3)	
C15	0.5156 (2)	0.9380 (2)	0.69937 (17)	0.0245 (4)	
H15A	0.4980	1.0404	0.6625	0.029*	
H15B	0.6326	0.9028	0.6600	0.029*	
C16	0.5090 (3)	0.9368 (3)	0.8421 (2)	0.0471 (6)	
H16A	0.3957	0.9768	0.8807	0.071*	
H16B	0.6030	0.9975	0.8603	0.071*	
H16C	0.5241	0.8353	0.8790	0.071*	

### Atomic displacement parameters (Å^2^)

**Table:**

	*U*^11^	*U*^22^	*U*^33^	*U*^12^	*U*^13^	*U*^23^
S1	0.0229 (2)	0.0171 (2)	0.0166 (2)	0.00254 (17)	−0.00388 (17)	−0.00395 (16)
C1	0.0247 (9)	0.0143 (8)	0.0237 (9)	0.0026 (7)	−0.0072 (7)	−0.0022 (7)
C2	0.0210 (9)	0.0167 (8)	0.0179 (8)	−0.0010 (7)	−0.0032 (7)	−0.0007 (7)
C3	0.0122 (8)	0.0167 (8)	0.0168 (8)	−0.0012 (6)	−0.0044 (6)	−0.0021 (6)
C4	0.0142 (8)	0.0158 (8)	0.0177 (8)	0.0005 (6)	−0.0046 (6)	−0.0038 (7)
C5	0.0120 (8)	0.0167 (8)	0.0185 (9)	−0.0017 (6)	−0.0051 (6)	−0.0023 (7)
O5	0.0252 (7)	0.0177 (6)	0.0184 (6)	0.0006 (5)	−0.0062 (5)	0.0014 (5)
C6	0.0115 (8)	0.0155 (8)	0.0189 (8)	0.0029 (6)	−0.0031 (6)	−0.0027 (7)
O6	0.0136 (6)	0.0150 (6)	0.0327 (7)	0.0042 (5)	−0.0057 (5)	−0.0029 (5)
C7	0.0128 (8)	0.0148 (8)	0.0198 (9)	−0.0012 (6)	−0.0022 (6)	−0.0008 (7)
C8	0.0174 (9)	0.0167 (8)	0.0169 (8)	−0.0037 (7)	0.0015 (7)	−0.0009 (7)
C9	0.0246 (9)	0.0243 (9)	0.0194 (9)	0.0031 (7)	−0.0023 (7)	−0.0050 (7)
C10	0.0317 (11)	0.0351 (11)	0.0220 (10)	0.0013 (9)	−0.0086 (8)	−0.0006 (8)
C11	0.0407 (11)	0.0329 (11)	0.0146 (9)	−0.0079 (9)	−0.0034 (8)	−0.0034 (8)
C12	0.0385 (11)	0.0207 (9)	0.0199 (9)	−0.0036 (8)	0.0070 (8)	−0.0066 (7)
C13	0.0234 (9)	0.0185 (9)	0.0212 (9)	−0.0008 (7)	0.0014 (7)	0.0001 (7)
C14	0.0163 (8)	0.0157 (8)	0.0116 (8)	−0.0006 (7)	−0.0003 (6)	−0.0006 (6)
O14	0.0220 (6)	0.0149 (6)	0.0270 (7)	0.0009 (5)	−0.0047 (5)	−0.0034 (5)
O15	0.0118 (6)	0.0222 (6)	0.0278 (7)	0.0012 (5)	−0.0050 (5)	−0.0098 (5)
C15	0.0139 (9)	0.0281 (10)	0.0334 (10)	−0.0044 (7)	−0.0040 (7)	−0.0113 (8)
C16	0.0419 (13)	0.0704 (16)	0.0341 (12)	−0.0203 (12)	−0.0160 (10)	−0.0069 (11)

### Geometric parameters (Å, °)

**Table:**

S1—C1	1.7164 (17)	C9—C10	1.395 (2)
S1—C4	1.7171 (16)	C9—H9	0.9500
C1—C2	1.365 (2)	C10—C11	1.376 (3)
C1—H1	0.9500	C10—H10	0.9500
C2—C3	1.417 (2)	C11—C12	1.384 (3)
C2—H2	0.9500	C11—H11	0.9500
C3—C4	1.374 (2)	C12—C13	1.389 (2)
C3—C7	1.509 (2)	C12—H12	0.9500
C4—C5	1.443 (2)	C13—H13	0.9500
C5—O5	1.2190 (19)	C14—O14	1.2041 (19)
C5—C6	1.560 (2)	C14—O15	1.3332 (19)
C6—O6	1.4018 (18)	O15—C15	1.4689 (19)
C6—C14	1.531 (2)	C15—C16	1.491 (3)
C6—C7	1.567 (2)	C15—H15A	0.9900
O6—H6	0.82 (2)	C15—H15B	0.9900
C7—C8	1.520 (2)	C16—H16A	0.9800
C7—H7	1.0000	C16—H16B	0.9800
C8—C9	1.389 (2)	C16—H16C	0.9800
C8—C13	1.395 (2)		
			
C1—S1—C4	89.88 (8)	C8—C9—C10	120.27 (16)
C2—C1—S1	113.94 (13)	C8—C9—H9	119.9
C2—C1—H1	123.0	C10—C9—H9	119.9
S1—C1—H1	123.0	C11—C10—C9	120.64 (17)
C1—C2—C3	111.12 (15)	C11—C10—H10	119.7
C1—C2—H2	124.4	C9—C10—H10	119.7
C3—C2—H2	124.4	C10—C11—C12	119.60 (16)
C4—C3—C2	112.06 (14)	C10—C11—H11	120.2
C4—C3—C7	112.16 (14)	C12—C11—H11	120.2
C2—C3—C7	135.47 (15)	C11—C12—C13	120.06 (16)
C3—C4—C5	112.25 (14)	C11—C12—H12	120.0
C3—C4—S1	112.99 (12)	C13—C12—H12	120.0
C5—C4—S1	134.64 (13)	C12—C13—C8	120.82 (17)
O5—C5—C4	130.13 (15)	C12—C13—H13	119.6
O5—C5—C6	124.00 (14)	C8—C13—H13	119.6
C4—C5—C6	105.87 (13)	O14—C14—O15	125.38 (15)
O6—C6—C14	109.60 (13)	O14—C14—C6	122.73 (14)
O6—C6—C5	111.05 (12)	O15—C14—C6	111.83 (13)
C14—C6—C5	106.85 (12)	C14—O15—C15	115.81 (12)
O6—C6—C7	110.20 (12)	O15—C15—C16	111.60 (15)
C14—C6—C7	113.48 (12)	O15—C15—H15A	109.3
C5—C6—C7	105.56 (12)	C16—C15—H15A	109.3
C6—O6—H6	111.8 (14)	O15—C15—H15B	109.3
C3—C7—C8	119.14 (13)	C16—C15—H15B	109.3
C3—C7—C6	102.88 (12)	H15A—C15—H15B	108.0
C8—C7—C6	114.66 (13)	C15—C16—H16A	109.5
C3—C7—H7	106.4	C15—C16—H16B	109.5
C8—C7—H7	106.4	H16A—C16—H16B	109.5
C6—C7—H7	106.4	C15—C16—H16C	109.5
C9—C8—C13	118.56 (15)	H16A—C16—H16C	109.5
C9—C8—C7	123.45 (14)	H16B—C16—H16C	109.5
C13—C8—C7	117.99 (15)		

### Hydrogen-bond geometry (Å, °)

**Table:**

*D*—H···*A*	*D*—H	H···*A*	*D*···*A*	*D*—H···*A*
O6—H6···O14	0.82 (2)	2.27 (2)	2.6705 (16)	110.8 (16)
O6—H6···O5^i^	0.82 (2)	2.06 (2)	2.7973 (16)	150.5 (19)
C1—H1···O14^ii^	0.95	2.56	3.157 (2)	121
C11—H11···O5^iii^	0.95	2.52	3.386 (2)	152
C15—H15B···O6^iv^	0.99	2.27	3.206 (2)	157
C7—H7···S1^v^	1.00	2.97	3.7855 (16)	139
C12—H12···O14^vi^	0.95	2.61	3.497 (2)	157

Symmetry codes: (i) −*x*, −*y*+2, −*z*+1; (ii) *x*, *y*−1, *z*; (iii) *x*, *y*, *z*+1; (iv) *x*+1, *y*, *z*; (v) −*x*, −*y*+1, −*z*+1; (vi) −*x*, −*y*+2, −*z*+2.
